# Evaluation of seven gene signature for predicting HCV recurrence post-liver transplantation

**DOI:** 10.1186/s43141-021-00266-4

**Published:** 2021-11-10

**Authors:** Ghada M. Salum, Mai Abd el Meguid, Tawfeek H. Abelhafez, Eman Medhat, Ashraf O. Abdel Aziz, Reham Dawood

**Affiliations:** 1 Department of Microbial Biotechnology, Genetic Engineering Division, National Research Centre, Dokki, P.O. 12622, Giza, Egypt; 2 Department of Endemic Medicine and Hepato-gastroenterology, Faculty of Medicine, Cairo University, Cairo, Egypt

**Keywords:** HCV, CRS, Donor steatosis, Recipients, Orthotropic liver transplantation, SNP, Allelic discrimination

## Abstract

**Background:**

Orthotropic liver transplantation (OLT) offers a therapeutic choice for hepatocellular carcinoma (HCC) patients. The poor outcome of liver transplantation is HCV recurrence. Several genome-wide associated studies (GWAS) have reported many genetic variants to be associated with HCV recurrence. Seven gene polymorphisms formed a cirrhosis risk score (CRS) signature that could be used to distinguish chronic HCV patients at high risk from those at low risk for cirrhosis in non-transplant patients. This study aims to examine the association of CRS score and other clinical parameters with the probability for HCC emergence and/or the rate of HCV recurrence following liver transplantation.

**Results:**

Seven gene polymorphisms, forming the CRS, were genotyped by real-time PCR using allelic discrimination protocol in 199 end-stage liver disease patients (79 child A, 43 child B, and 77child C), comprising 106 patients who encountered liver transplantation. Recipient CRS scores were correlated with HCV recurrence (HCV-Rec) at the end of the third year after OLT. Around 81% (39) recipients with low steatosis (LS; < 3.5%) donor percentage revealed no HCV recurrence (non-Rec) (*p*<0.001). CRS score could distinguish between child A, child B, and child C only at the low-risk group. Among the HCV Rec group 27% (8/30), 40% (12/30), and 33% (10/30) fell into the high, moderate, and low CRS risk groups, respectively. Stepwise logistic regression evinced two features more likely to be seen in HCV-Rec patients: abnormal ALT [OR, 1.1; 95% CI, 1.02–1.2] and donor steatosis >3.5% [OR, 46.07; 95% CI, 1.5–1407.8].

**Conclusions:**

Accordingly, the CRS score seems to be less useful to predict HCV recurrence after OLT. ALT and donor steatosis (exceed 3.5%) can significantly promote the HCV recurrence post-OLT. Moreover, the combination of MMF and CNI positively heightens HCV recurrence.

**Supplementary Information:**

The online version contains supplementary material available at 10.1186/s43141-021-00266-4.

## Background

Despite the incredible progress in treating hepatitis C virus (HCV), many patients are still at the risk of disease progression to cirrhosis and hepatocellular carcinoma (HCC) at different rates [1–3]. Our country launched “100 million lives” campaign declaring that viral hepatitis should be eliminated by 2030. Elimination of HCV will confer economic benefits and substantial health and, most critically, the avoidance of above 1.2 million deaths yearly [4]. The Child-Pugh score has been used as a prognostic predictor of postoperative mortality and has been taken into account in a number of staging systems [5, 6]. To date, surgery remains the master prognostic tool for the long-term survival of HCC patients; nevertheless, HCC is frequently associated with chronic viral hepatitis and over 80% of tumors are unresectable [7, 8]. OLT submits a therapeutic choice for HCC patients, particularly in cirrhotic patients without distant metastasis of HCC.

Nonetheless, the main potential cause for the poor outcome of liver transplanted patients post-OLT is HCV recurrence [9]. Recurrent HCV-associated liver disease leads to a consequent loss of graft in about one third of patients within 5 years of OLT and recurrent HCV-associated graft failure is the main cause of patient mortality and re-transplantation in the 5th postoperative year [10]. Several factors are crucial to minimize the complications and improve the clinical outcome such as choice of a suitable donor, appropriate immunosuppressive treatment, and genetic risk stratification prior to transplantation [11, 12].

Data on the genetic risk of HCV recurrence post-liver transplantation are scarce [13]. Recent data on Toll-like receptor (TLR)-related genes have recorded an augmented risk of HCC recurrence for donor’s TLR4 (rs1927914) and recipient’s TLR9 (rs187084) and IL-15 (rs10519613) polymorphisms, respectively [14–16]. Additionally, the model for end-stage liver disease (MELD), AST to platelet ratio index (APRI), and fibrosis scoring system (FIB-4) was used to evaluate liver fibrosis post-LT for HCV-related liver disease [17], while some skepticism was stated about these scores [18].

Up to date, no study delineate-specific predictive biomarkers of HCV recurrence in post-transplant patients [19]. Cirrhosis Risk Score (CRS) is a polygenic signature firstly defined by Huang [20] and stratified the cirrhosis risk in many populations better than clinical factors [20, 21]. CRS is relying on a set of seven single-nucleotide polymorphisms (SNPs) in six genes: AP3S2, AQP2, AZIN1, STXBP5L, TLR4, TRPM5, and in the intergenic region between DEGS1 and NVL (see Table 1).

**Table 1:** General details on the 7 candidate SNPs in the CRS score

Genes	Gene name	rs number	Chromosome	Gene biological functions
**AZIN1**	Antizyme inhibitor	rs62522600	**(Chr8)**	Polyamine biosynthesis, cell proliferation [22]
**TLR4**	Toll-like receptor 4	rs4986791	**(Chr9)**	Important pathogen recognition receptors [23]
**TRPM5**	Transient receptor potential cation channel subfamily member 5	rs886277	**(Chr11)**	Taste responses, specific physiological function in liver is unknown [24].
**AP3S2**	Adaptor-related protein complex 3 sigma 2 subunit	rs2290351	**(Chr15)**	Unknown
**DESGS1**	Degenerative spermatocyte homolog1 lipid desaturase	rs4290029	**(Chr1)**	Lipid metabolism and transport, cell growth [25]
**STXBP5L**	Syntaxin binding protein	rs17740066	**(Chr3)**	Inhibits endothelial exocytosis [26]
**AQP2**	Aquaporin 2	rs2878771	**(Chr12)**	Water reabsorption, vasopressin regulation [27]

*SNP* single-nucleotide polymorphism, *rs* accession number on databases referring to specific SNPs

Theoretically, CRS may be used to stratify patients who are eligible for OLT or not better than a liver biopsy. The latter represents a single time point in the extended natural history of chronic infection, while genetic markers are and “life-long.” Also, we have a growing base of evidence linking a variant in the IL6 rs1800795 G allele with HCV recurrence post-LT [28]. Moreover, we finished a promising study which validated CRS performance in 240 Egyptian HCV-infected patients with different fibrosis grades [29]. Herein, to fuel the novel debate on Child-Pugh score, we assess it with a CRS signature (as an intrinsic genetic marker). Moreover, we aim to validate a CRS signature for recipients to assess the risk for HCV recurrence following OLT in Egyptian liver transplanted patients as it may serve as an early noninvasive genetic biomarker for HCV recurrence post-OLT.

## Methods

### Study design

The study included a total of 199 end-stage liver disease patients (79 child A, 43 child B, and 77 child C); comprising 106 patients who encountered OLT. All patients encountered orthotopic LT for HCV. The histologic degree of macrovesicular steatosis was determined. Patients suffering from acute rejection episodes were excluded. The primary immunosuppressive for all patients consisted of a calcineurin inhibitor (CNI) with or without Mycophenolate mofetil (MMF) or Everolimus at the second year (according to specific side effects or renal function). In this study, the patients were divided into two groups according to the HCV recurrence: group 1, HCV Rec group (*n*=32) and group 2, non-Rec group (*n*=48).

All patients were also evaluated by clinical and laboratory parameters, including biochemical (alanine aminotransferase (ALT), aspartate aminotransferase (AST), albumin, bilirubin total, and platelets count (Plt)), and serological test (anti-HCV) and histopathology of liver biopsy. The diagnosis of HCC was made after reviewing images generated with several imaging modalities. Patients having other cancers were excluded.

### Liver biopsy evaluation

Liver biopsy was operated for all recipients at the end of the third year following primary OLT. Liver biopsies were evaluated by a pathologist who was unaware of clinical and demographic data that were obtained. Fibrosis stages were defined using the METAVIR scoring system and categorized according to F0: none, F1: portal widening, F2: bridging fibrosis, and F3: bridging fibrosis with lobular distortion. We also stratified the fibrotic patients based on the inflammation activity into A1, A2, A3 refer to mild, moderate, and severe, respectively.

### Extraction of peripheral blood DNA

The peripheral blood on EDTA was withdrawn from all subjects, and genomic DNA was extracted using genomic DNA extraction kits (Qiagen, Milan Italy). Purified genomic DNA samples were quantified using ultraviolet absorbance at 260 nm using a Thermo Scientific NanoDrop™ Spectrophotometer. The DNA was stored at −20°C.

### Cirrhosis risk signature (CRS) genotyping

The 7 SNPs identified previously by Huang et al. [20] were genotyped using a real-time PCR protocol based on the pre-validated TaqMan MGB^TM^ probe for allelic discrimination assay (Applied Biosystems). Briefly, 1.25 μL of a 40X combined primer and probe mix (ABI/Life Technologies, USA) was added to 12.5 μL of 2X TaqMan® Universal PCR master mix (ABI/Life Technologies, USA) in a 25-μL final volume of DNAse/RNAse-free water (Invitrogen/Life Technologies, USA) and template. The cycle conditions were 95 °C for 10 min, 95 °C for 15 s, and 60 °C for 1 min. The last two steps were repeated 40 times. The PCR run was performed on Rotor-Gene real-time PCR system (Qiagen, Santa Clarita, CA). Allelic discrimination plots were produced in Statistical Package for The Social Sciences (SPSS version 16.0; SPSS, Chicago, IL).

In this study, we consciously used the classification launched in the original publication by Huang et al. [20]: a CRS > 0.7 signifies patients with a high risk of advanced liver fibrosis, CRS < 0.5 signifies a low risk of fibrosis, a CRS of 0.5 to 0.7 signifies an intermediate risk, and upon the score the patient was assigned to appropriate risk category.

### Statistical analysis

Data were analyzed using SPSS 16.0. Data were presented as mean ±standard deviation. Categorical variables were compared with the *χ*^2^ or Fisher’s exact tests, each when appropriate, and the effect of differences was established by calculating the odds ratio with the 95% confidence interval (95%CI). According to variable distribution, one-way ANOVA or nonparametric Kruskal–Wallis test was used for multi-group comparisons. The nonparametric Mann–Whitney *U* test was used to compare median values between two groups for quantitative data. A difference between groups was significant if *P*< 0.05.

## Results

### Description of the study patients

Our study started on 199 end-stage liver disease patients that were categorized into 79 Child-Pugh class A, 43 child B, and 77 child C. Male patients are represented 75% from child A, 81% from child B, and 75% from child C (*p*=0.7). Patients’ baseline characteristics are represented in Table 2. Medical data records allowed a follow-up of only 120 patients who were eligible for liver transplantation, see Table 2.

**Table 2:** Clinical data of the 199 end-stage liver disease (Child-Pugh class A, child B, and child C)

	End-stage liver disease patients			
	Child A	Child B	Child C	***P*** value
	*N*=79	*N*=43	*N*=77	
**Age**	60 a (55–64)	55b (51–60)	52c (48.50–57)	<0.001 HS
**Hb.%**	12.5 a (11–13.70)	12 b (10.70–13)	12b (11–13)	0.031
**WBCsX10**^**3**^	5.30 a (4.30–6.50)	5.10 a (4–6)	5.60 a (4–6.95)	0.6
**Platelets**X10^3^	130 b (96–160)	156 ab (90–180)	165 a (121–210)	<0.001 HS
**ALT**	43 a (34–50)	40 a (33–47)	46 a (30–110)	0.09
**AST**	52 a (45–63)	48 a (29–65)	46 a (21.5–86.5)	0.509
**Alb.**	3.50 b (3.20–3.80)	3.40 b (2.80–3.80)	3.80 a (3.45–4.00)	<0.001 HS
**Creat.**	1.00 a (0.90–1.10)	1.00 a (0.90–1.10)	1.00 a (0.90–1.10)	0.86

Data are expressed as median (IQR). *Hb.* states to hemoglobin, *ALT* states to serum alanine aminotransferase, and *AST* states to serum aspartate aminotransferase, *Alb.* states to albumin

Identical letters in rows mean no significant difference at the level of 0.05

Different letters in rows mean significant difference at the level of 0.05

HS means highly significant

### Genotyping of the seven genes

**Fig. 1:**
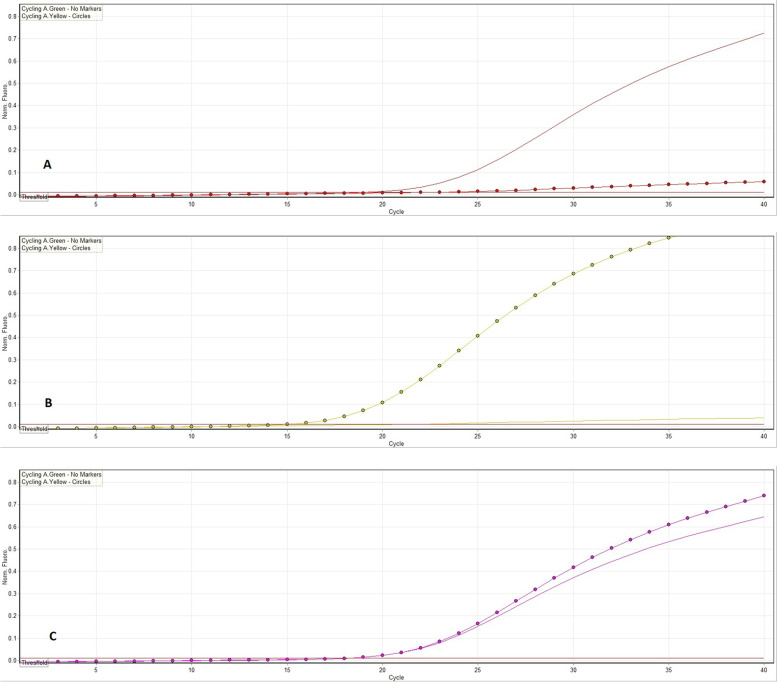
Allelic discrimination curves produced by Rotor Gene real-time PCR system. The x -axis represents the amplification cycle number and the y -axis represents the fluorescent value. (**A**) Example of homozygote alleles labeled with FAM™. (**B**) Example of homozygote alleles labeled with VIC™. (**C**) Example of heterozygote alleles (one labeled with FAM™ and the other labeled with VIC™)

Individual 7 candidate SNPs included in the genetic risk score (CRS) for each patient were listed in the S1 table. Some of the allelic discrimination results obtained from the real-time PCR for some genes were represented in Fig. 1

Previously, Huang et al. selected the seven genes that are involved in the cirrhosis prediction and evaluated the probability of each genotype in the cirrhotic and non-cirrhotic patients in the Caucasian population. Their findings were tabulated in Table 3 which illustrates that each SNP can take the value 0 or 1 based on the obtained genotype, and then, each value has two probabilities (assuming that the patient can be cirrhotic or non-cirrhotic). Each SNP was calculated independently of other SNPs.

**Table 3:** CRS algorithm deduced by Huang et al. [20]

Marker	Gene	SNP value=1	SNP value=0	***P***(snp=1l cirrhosis)	***P***(snp=1l no cirrhosis)	***P***(snp=0l cirrhosis)	***P***(snp=0l no cirrhosis)
**SNP 1**	**AZIN1 (Chr8)**	GG	GA, AA	0.928030303	0.801282051	0.071969697	0.198717949
**SNP 2**	**TLR4 (Chr9)**	CC	CT, TT	0.928301887	0.810126582	0.071698113	0.189873418
**SNP 3**	**TRPM5 (Chr11)**	TT	TC, CC	0.318181818	0.487341772	0.681818182	0.512658228
**SNP 4**	**(AP3S2) (Chr15)**	GG	GA, AA	0.554716981	0.696202532	0.445283019	0.303797468
**SNP 5**	**none (Chr1)**	GG	GC, CC	0.78490566	0.610062893	0.21509434	0.389937107
**SNP 6**	**(STXBP5L)(Chr3)**	GG	GA, AA	0.78490566	0.905660377	0.21509434	0.094339623
**SNP 7**	**AQP2 (Chr12)**	GG	GC, CC	0.747169811	0.578616352	0.252830189	0.421383648

The values obtained from Table 3 were substituted in the following Naïve Bayes formula:

$$\mathrm{CRS}=\frac{0.626\ast P\;\left(\left.\mathrm{S}\;\right|\;\mathrm{cirrhosis}\right)}{0.626\ast P\left(\left.S\;\right|\; cirrhosis\right)+0.374\ast P\;\left(\left.\mathrm{S}\;\right|\;\mathrm{no}\;\mathrm{cirrhosis}\right)}$$

*P* (S│cirrhosis) and *P* (S│no cirrhosis) referred to the estimated probabilities of (cirrhotic and non-cirrhotic) patients, respectively.

In the current study, we followed the same steps to build up the CRS value and to evaluate the validity of this formula in the Egyptian population.

The detailed calculation method was shown in Huang et al. [20].

### CRS score could expect the probability for HCC emergence

Particularly, patients were categorized into 3 groups based on their CRS values. Patients were either at high risk (CRS7 > 0.7), intermediate risk (CRS7 0.5–0.7), or at low risk of cirrhosis (CRS7 < 0.5).

The distribution of CRS value was different in child A (21.5% low risk, 26.6% intermediate risk, and 51.9% high risk) than in child B (25.6% low risk, 23.3% intermediate risk, and 51.2% high risk) (*p* =0.052).

The median of CRS values of patients at low risk differed significantly between child A (0.42; 0.39–0.44), child B (0.39; 0.26–0.4), and child C (0.39; 0.26–0.4) (*p* =0.009). While intermediate risk patients were differed between child A (0.6; 0.59–0.6), child B (0.6; 0.56–0.62), and child C (0.6; 0.56–0.67) (*p* =0.8). Similarly, high-risk group median varied between child A (0.77; 0.74–0.86), child B (0.77; 0.74–0.86), and child C (0.77; 0.75–0.8) (*p* =0.59; see Table 4).

**Table 4:** Frequency of children A, B, and C among CRS categories

		Child A	Child B	Child C	***P*** value
**CRS score**	**Low**	0.416 a (0.39–0.44)	0.39 b (0.26–0.40)	0.39 b (0.26–0.40)	0.009 s
	**Intermediate**	0.612 a (0.59–0.61)	0.60 a (0.56–0.62)	0.612 a (0.56–0.67)	0.831 ns
	**High**	0.772 a (0.74–0.86)	0.772 a (0.74–0.86)	0.772 a (0.75–0.81)	0.598 ns

Identical letters in rows mean no significant difference at the level of 0.05

Different letters in rows mean significant difference at the level of 0.05

s means significant

ns means not significant

Importantly, we also stratified the patients based on the occurrence of clinically evident HCC or not; the CRS median for patients who progressed to HCC was 0.62 (0.39–0.77) and while the CRS median for patients without HCC was 0.59 (0.35–0.70) (*p*=0.044).

### Description of the liver transplantation patients

From all the 106 patients, the recipient’s score was available in 80 patients (CRS could not be calculated for the sake of technical complexity such as amplification failure of one the SNP reaction). Among 80 consecutive recipients, 78% were male and 22% female, with a mean age of (50.2±7.3) ranging between 23 and 60 years. The mean age for the HCV recurrence group (HCV-Rec) was 50.44 ± 7.2, and it was 50.06 ± 7.5 (*p* = 0.8) for the non-Rec group. The male to female ratio (M/F) was (23/9) in the HCV Rec group and (39/9) in the non-Rec group (*p* = 0.3; see Tables 5 and 6).

**Table 5:** Biochemical parameters of the 106 recipients post-OLT

	Liver transplanted patients				
	HCV recurrence		Non-recurrence		***P*** value
	Mean	SD	Mean	SD	
**BMI**	27.41	2.41	26.83	2.44	0.339
**Hb.%**	11.25	1.18	11.74	1.13	0.077
**WBCs**X10^3^	4.92	1.48	6.19	1.68	0.001
**Platelets**X10^3^	146.00	38.60	187.52	33.16	< 0.001
**ALT**	125.87	46.15	36.52	27.53	< 0.001
**AST**	97.59	40.41	27.87	21.15	< 0.001
**ALP**	141.28	58.55	73.79	65.87	< 0.001
**GGT**	80.50	21.07	58.63	77.47	< 0.001
**Alb.**	3.69	0.30	3.92	0.28	0.002
**T.Bil.**	2.11	0.88	1.16	0.66	< 0.001
**D.Bil.**	1.18	0.66	.52	0.52	< 0.001
**Urea**	42.56	10.61	41.10	13.46	0.252
**Creat.**	1.04	0.32	1.03	0.25	0.858
**Na**	139.06	2.03	138.92	2.57	0.968
**K**	3.85	0.19	4.00	0.30	0.004

Data are expressed as mean ±standard deviation

*BMI* body mass index, *Hb* hemoglobin, *ALT* serum alanine aminotransferase, *AST* serum aspartate aminotransferase, *T.Bil & D.Bil* total and direct bilirubin, respectively

**Table 6:** Clinical features of the 106 recipients post-OLT

	Liver transplanted patients				
	HCV recurrence		Non-recurrence		***P*** value
	Mean	SD	Mean	SD	
**Age**	50.44	7.22	50.06	7.48	0.771
**MELD**	17.12	1.88	17.15	1.86	0.913
**Age of donor**	30.94	5.86	29.90	4.44	0.335
**Donor steatosis %**	3.81	2.82	2.23	2.09	0.009
**HCV RNA before (IU/ml)**	564.05	382.79	571.26	527.23	0.57
**HCV RNA post (IU/ml)**	1806.20	918.0	120.97	888.48	0.002

*IU/ml* international unit per ml

Patients with HCV-Rec had statistically significant higher levels of AST, ALT, GGT, ALP, total and direct bilirubin (*p*_for all_<0.001), and significantly lower potassium (*p*=0.004), platelet count (*p*=0.001), WBCs count (*p*=0.001), and albumin level (*p*= 0.002), as compared to those in the no HCV recurrence (non-Rec) group. Also, urea, sodium, BMI, and creatinine levels were slightly elevated but without reaching significance. MELD score did not display any variance between the two studied groups.

To examine the role of pre and/or post-operative levels of HCV RNA on HCV-Rec frequency; each level was correlated independently with recurrence. Pre-OLT serum HCV RNA levels did not reveal any correlation (*p*=0.8). On the contrary, serum HCV-RNA levels post-OLT were 1806.20 ± 918.02 IU/mL in the HCV-Rec group and 1280.97 ± 888.48 IU/mL in the non-Rec group. The mean loads of serum HCV-RNA levels after OLT were significantly related with HCV Rec (*p* = 0.002; see Table 6).

Upon categorizing patients according to the immune suppressive regimen, 24 (77.4%) of HCV-Rec were treated with CNI plus MMF, 4 (13%) were treated with CNI plus Everolimus, and 3 (10%) were treated only with CNI. On the other side, 13 (27%) of the Non-Rec group were treated with CNI plus MMF, 9 (19%) were treated with CNI plus Everolimus, and 26 (54%) were treated with CNI only. Clearly, CNI plus MMF regimen was significantly found in the HCV Rec group (*p*<0.001).

### Detection of the HCV recurrence according to donor steatosis percentage

To gain viewpoints on the impact of donor steatosis on HCV recurrences after transplantation, patients were grouped according to donor steatosis percentage into patients who have high steatosis (HS; > 3.5) and patients who have low steatosis (LS; < 3.5). Around 81% (39) patients have non-Rec were LS, while 61% (19) patients suffered HCV Rec were HS. On the other hand, 39% (12) patients have HCV Rec were LS, while 19% (9) patients have HCV Rec were HS (*p*<0.001; see Table 7).

**Table 7:** Frequency of HCV recurrence among CRS categories; donor steatosis groups. CRS subgroups are high risk, CRS > 0.7; moderate risk, CRS 0.5–0.7; low risk, CRS < 0.5

		Liver transplanted patients				***P*** value
		HCV recurrence		Non-recurrence		
		Count	%	Count	%	
**CRS SCORE**	**Low risk**	10	33.3%	15	34.1%	0.943
	**Moderate risk**	12	40.0%	16	36.4%	
	**High risk**	8	26.7%	13	29.5%	
**Donor steatosis %**	**Low<3.5**	12	38.7%	39	81.3%	<0.001
	**High>3.5**	19	61.3%	9	18.8%	

### Detection of the HCV recurrence according to CRS score

According to the CRS score suggested by Huang et al., the patients were stratified into three risk subgroups (high risk, CRS > 0.7; moderate risk, CRS 0.5–0.7; low risk, CRS < 0.5). To rule whether the CRS score could discriminate between patients who experienced the HCV-Rec group versus patients with non-Rec, the distribution of the CRS score was compared among the two groups. Among the HCV-Rec group, 27% (8/30), 40% (12/30), and 33% (10/30) fell into the high, moderate, and low CRS risk groups, respectively. While among the non-Rec group, 30% (13/44), 37% (16/44), and 34% (15/44) fell into the high, moderate, and low CRS risk groups, respectively. Unfortunately, the association between CRS score subgroups and the HCV Rec did not reach the statistical significance (*p*<0.9; see Table 6).

Notably, the CRS values cannot predict the HCV recurrence. Even though only 33.3% of the patients (10/30) with a CRS < 0.5 and 40% of the patients (12/30) with a CRS of 0.5 to 0.7, 27% of the patients (8/30) with a CRS > 0.7 suffered HCV-Rec (*p* =0.9). Importantly, the median of the CRS score was not significantly different between HCV-Rec and non-Rec patients (median=0.6 for both groups; *p*=0.4).

### Detection of the severity of inflammation according to CRS score

To examine the potential role of the CRS on the hepatic inflammation, patients were gathered into mild (A0F0-A1F1) and advanced inflammation (A2F2-A3F3) groups. Overall, 40% of the transplant patients progressed to at least A2F2 during follow-up, whereas 60% of the subjects were between A0F0 to A1F1. Around 28% (8/29) patients of (A2F2-A3F3) group met the high-risk CRS score, while 31% (9/29) patients met the low-risk CRS score. On the contrary, 29% (13/45) patients of (A0F0-A1F1) group met the high-risk CRS score, while 36% (16/45) patients met the low-risk CRS score (*p*=0.9).

### Detection of the severity of inflammation according to donor steatosis

To show the impact of donor steatosis on the recipient’s inflammation progression, around 61% (19/29) of recipients have a donor with HS progressed to at least A2F2 during follow-up, whereas 19% (9/45) of the recipients have mild inflammation (A0F0 to A1F1). Around 81% (39/45) of recipients have a donor with LS revealed mild inflammation (A0F0 to A1F1) and 39% (12/29) of recipients progressed to at least A2F2 (*p*<0.001).

### CRS scoring using new cutoff for HCV recurrence prediction

Our latest study on 400 HCV infected patients with different fibrosis grades concluded that our best CRS cutoff value appraised from roc curve analysis is 0.59 (under publication), accordingly the patients of the current study were regrouped into low risk with a CRS < 0.59 and high-risk patients with a CRS above the mentioned cutoff. The patients who suffered HCV Rec represented 57% (17/30) of high-risk CRS >0.59 but 41% (18/44) of non-Rec patients met low CRS < 0.59 (*p* =0.8).

### Stepwise logistic regression analysis

When Stepwise logistic regression was applied to the baseline data, three features were more likely to be effective in HCV rec patients’ more than non-rec: abnormal ALT [odds ratio (OR), 1.1; 95% confidence interval (CI), 1.02–1.2] and donor steatosis >3.5% (OR, 46.07; 95% CI, 1.5–1407.8; see Table 7). The results of this analysis are depicted in Table 8. The CRS was not an independent predictor of HCV-Rec.

**Table 8:** Stepwise logistic regression

		***B***	S.E.	***P*** value	OR	95% C.I.	
						Lower	Upper
**HCV recurrence**	**ALT**	0.103	0.039	0.008	1.109	1.028	1.196
	**Donor steatosis % (<3.5)**	3.830	1.745	0.028	46.069	1.508	1407.848

*S.E* standard error *OR* odds ratio *CI* confidence interval

## Discussion

In HCV-related hepatic cirrhosis, hepatocellular carcinoma (HCC) occurs at an annual rate of about 3% [30]. Orthotropic liver transplantation (OLT) offers a treatment option for end-stage liver disease patients. HCV reinfection is nearly common after OLT, and it is estimated that up to 70% of patients will undergo histologic chronic hepatitis C [31], with a greater risk of graft rejection relative to recipients who are transplanted for other etiologies.

It is noteworthy that genetic data will be used to assess the disease risk, with possible therapeutic benefits [14, 32]. CRS score successfully differentiated chronic HCV patients with high risk versus those with low risk for cirrhosis better than clinical factors [20].

We currently examined the association of the CRS score with the probability for HCC emergence and/or the rate of HCV recurrence following liver transplantation. Theoretically, each of the seven most predictive markers provided only moderate predictability, whereas the combination of these 7 SNPs seems to be robust and predictive. The median of the CRS score significantly differentiates patients with clinically evident HCC from patients who did not progress to HCC. The median of the CRS score was significantly different between child A, child B, and child C only at a low-risk group. New researches warrant that Child-Pugh score usage as a risk prediction tool should be revisited [33]. On the other side, the median of the CRS score was not significantly different between HCV recurrence and non-recurrence patients. Accordingly, results in our cohort tackled that CRS cannot predict the HCV recurrence after OLT. However, a recent study shed the light on the clinical significance of the CRS genotype in the donor organ and revealed a strong association between the donor CRS and early fibrosis progression after OLT, especially in HCV-negative patients [34]. It is worth noting that the coinfection with other viruses triggers the cellular apoptosis and accelerating the HCC development. Therefore, early diagnosis of cirrhosis is crucial to avoid the mortality associated with HIV. Fernández-Rodríguez et al. reported that the diagnostic value of the CRS to deduce the liver fibrosis deterioration is limited in HIV/HCV coinfected patients [35]. Other well-known genetic variations as IL1B and IL28B evinced a statistically significant correlation with the poor outcome post-transplantation [12].

Liver function tests were repetitively delineated to affect HCV recurrence after OLT [10, 12]. Definitely, our data showed that the increased risk of the HCV recurrence was correlated with augmented ALT, AST, and ALP levels. Feurer et al. [36] negated the correlation between liver function serum levels and disease-free survival rates. Our data shed light that HCV viral load post-transplant is significantly affecting HCV recurrence. Supportive studies affirmed that advanced donor age and/or high HCV viral loads post-transplant corresponded with aggressive HCV recurrence and allograft loss in HCV-positive liver transplant recipients [12, 37].

Cyclosporine (as a calcineurin inhibitor (CNI))-based regimen is the main immuno-suppression protocol followed in this study. This regimen was accompanied by mycophenolate mofetil (MMF) or Everolimus. CNI is supposed to bind to the cytosolic protein cyclophilin (an immunophilin) of T-lymphocytes [38]. Mycophenolic acid acts as a selective and reversible inhibitor of Inosine-5′-monophosphate dehydrogenase, whereas Everolimus is an inhibitor of mammalian target of rapamycin (mTOR) [39]. In our study, the addition of MMF to CNI positively heightens HCV recurrence rate while Everolimus did not negatively alter its rate which was supported by former studies [40].

Decisively, the outcome is better once a proper selection of patients is performed [10]. However, many surgeons have shown an augmented risk for inferior post-transplant outcomes in case of donor livers with moderate or severe large droplet macrosteatosis (ld-MaS), although donor livers with small droplet macrosteatosis (sd-MaS) or mild (<30%) ld-MaS are safe for transplantation [41, 42]. The combined analysis confirmed that the degree of steatosis in donors’ livers was below 3.5% avoiding the possibility of having a worse outcome. Donor liver steatosis impacts graft function, long-term consequences of the recipient allograft, and donor hepatic recovery. Indeed, transplanting a steatotic liver may lead to reperfusion injury/ischemia that may progress to an advanced rate of early graft dysfunction. Safe cutoff for transplantation range from 10 to 30% in accordance with the transplantation center regulation [43, 44].

Our study is limited by the small sample size (due to sample scarcity), the absence of the donor genotype and/or donor with macrovesicular steatosis of 30% or greater as the criterion for better comparison. No doubt that the donor genotype may synergy the genotype-phenotype association. On other point of view, the recipient genotype is more feasible and obtainable before transplantation earlier than the donor genotype. However, many studies correlated only the recipient genotype (for TLR4, IL6, and IL-28B SNPs) with the HCV recurrence [28, 41, 45, 46], and more attention is needed to identify new predictors.

## Conclusions

Based on our results, the prognostic value of the donor steatosis on HCV recurrence holds true in Egyptian CHC patients. Regression analysis showed that donor steatosis and ALT can significantly promote the HCV recurrence post-OLT. Because of the lack of significance, Child-Pugh score usage as a prognostic tool needs to be reassessed. Moreover, it is unlikely that CRS may be applicable in predicting the probability of HCV recurrence after OLT.

## Supplementary Information

**Additional file 1:** **Table S1**. Individual 7 candidate SNPs included in genetic risk score (CRS) and their calculated CRS score for the liver transplantation cohort.

## Data Availability

All relevant data are within the paper.

## Abbreviations

OLT: Orthotropic liver transplantation
CRS: Cirrhosis Risk Score
HCV: Hepatitis C virus
HCC: Hepatocellular carcinoma
MMF: Mycophenolate mofetil
CNI: Calcineurin inhibitor
TLR: Toll-like receptor
MELD: End-stage liver disease
APRI: Platelet ratio index
FIB-4: Fibrosis scoring system
ALT: Alanine aminotranferase
AST: Aspartate aminotransferase
PLT: Platelet
RT-PCR: Real-time polymerase chain reaction
SNPs: Single-nucleotide polymorphisms
SPSS: Statistical Package for the Social Sciences
WBCs: White blood cells
