# Supplementary material for: Evaluation of seven gene signature for predicting HCV recurrence post-liver transplantation
Source: J Genet Eng Biotechnol. 2021 Nov 10;19:174. doi: 10.1186/s43141-021-00266-4 (PMC8581076; doi:10.1186/s43141-021-00266-4)
Supplement: Supplementary file 1 — Additional file 1: Table S1. Individual 7 candidate SNPs included in genetic risk score (CRS) and their calculated CRS score for the liver transplantation cohort. [file 43141_2021_266_MOESM1_ESM.docx]

**Supporting Information**

**S1 Table**: Individual 7 candidate SNPs included in genetic risk score (CRS) and their calculated CRS score for the liver transplantation cohort.

| Sample NO. | HCV Recurrence | SNP1 | SNP2 | SNP3 | SNP4 | SNP5 | SNP6 | SNP7 | CRS SCORE |
| --- | --- | --- | --- | --- | --- | --- | --- | --- | --- |
| 1 | NO | GG | CC | CC | GA | GG | GG | GG | 0.86178178 |
| 2 | NO | GG | CC | CC | GG | GG | GG | GG | 0.772175621 |
| 5 | YES | GG | CC | CC | GG | GG | GG | GG | 0.772175621 |
| 3 | NO | GG | CC | TT | GG | GC | GG | CC | 0.248944786 |
| 4 | NO | GG | CC | CC | GA | GG | GG | GG | 0.861781 |
| 26 | NO | GG | CC | CC | GG | GC | GG | CC | 0.391173048 |
| 6 | NO | GG | CC | CC | GA | GG | GG | GG | 0.86178178 |
| 7 | YES | GG | CC | CC | GG | GG | GG | GC | 0.611627497 |
| 11 | NO | GG | CC | CC | GG | GC | GG | CC | 0.391173048 |
| 12 | NO | GG | CC | CC | GG | GG | GG | GG | 0.772176 |
| 13 | NO | GG | CC | CC | GG | GG | GG | GG | 0.772175621 |
| 14 | NO | GG | CC | CC | GG | GC | GG | GG | 0.59235918 |
| 15 | NO | GG | CC | CC | GG | GG | GG | GC | 0.611627497 |
| 16 | NO | GG | CC | CC | GG | GG | GG | GG | 0.772175621 |
| 17 | NO | GG | CC | CC | GG | GG | GG | CC | 0.611627497 |
| 18 | NO | GG | CC | CC | GG | GG | GG | GG | 0.727754221 |
| 19 | NO | GG | CC | CC | GG | GG | GG | GG | 0.727754221 |
| 20 | YES | GG | CC | CC | GG | GG | GG | GC | 0.611627497 |
| 21 | NO | GG | CC | CC | GG | GC | GG | CC | 0.403055056 |
| 22 | NO | GG | CC | CC | AA | GC | GG | GG | 0.72395056 |
| 23 | YES | GG | CC | CC | GG | GC | GG | GG | 0.59235918 |
| 24 | YES | GG | CC | CC | GG | GG | GG | GG | 0.727754221 |
| 25 | NO | GG | CC | CC | GA | GC | GG | GG | 0.258857 |
| 27 | NO | GG | CC | TT | GA | GC | GG | GG | 0.651791 |
| 28 | NO | GG | CC | CC | GA | GG | GG | CC | 0.6726 |
| 29 | NO | GG | CC | CC | GA | GG | GG | CC | 0.6726 |
| 30 | NO | GG | CC | TT | GG | GG | GG | GC | 0.488409629 |
| 31 | NO | GG | CC | TT | GG | GG | GG | GC | 0.488469 |
| 32 | YES | GG | CC | TT | GG | GG | GG | GC | 0.488409629 |
| 33 | NO | GG | CC | TT | GG | GC | GG | CC | 0.248944 |
| 35 | NO | GG | CC | CC | GA | GC | GG | GG | 0.258857 |
| 38 | NO | GG | CC | CC | GA | GC | GG | GG | 0.258857 |
| 40 | NO | GG | CC | TT | AA | CC | GG | GC | 0.372806547 |
| 41 | YES | GG | CC | CC | GA | GC | GG | GG | 0.258857 |
| 42 | NO | GG | CC | CC | GA | GG | GG | CC | 0.6726 |
| 50 | YES | GG | CC | CC | GA | GG | GG | CC | 0.6726 |
| 51 | YES | GG | CC | CC | GA | GG | GG | GG | 0.527616 |
| 52 | YES | GG | CC | CC | GA | GG | GG | CC | 0.6726 |
| 53 | YES | GG | CC | CC | GG | GC | GG | CC | 0.391173048 |
| 54 | NO | GG | CC | CC | GA | GG | GG | CC | 0.6726 |
| 55 | YES | GG | CC | CC | GG | GC | GG | CC | 0.391173048 |
| 60 | NO | GG | CC | CC | GG | GG | GG | GG | 0.772175621 |
| 64 | NO | GG | CC | CC | GG | GG | GG | GG | 0.772175621 |
| 65 | YES | GG | CC | CC | GG | GC | GG | CC | 0.403055056 |
| 66 | YES | GG | CC | CC | AA | GC | GG | GG | 0.727754221 |
| 67 | NO | GG | CC | CC | GA | CC | GG | GC | 0.553983677 |
| 69 | YES | GG | CC | CC | GA | GG | GG | GG | 0.86178178 |
| 70 | YES | GG | CC | CC | AA | GG | GG | CC | 0.743395056 |
| 71 | NO | GG | CC | CC | GG | GG | GG | GC | 0.611627497 |
| 72 | NO | GG | CC | TT | GA | GC | GG | GG | 0.56983677 |
| 73 | NO | GG | CC | CC | GG | GG | GG | CC | 0.611627497 |
| 74 | NO | GG | CC | CC | GA | GG | GG | GG | 0.86178178 |
| 75 | YES | GG | CC | CC | GA | GC | GG | GG | 0.258857 |
| 76 | YES | GG | CC | TT | GG | GG | GG | GG | 0.624604996 |
| 68 | NO | GG | CC | CC | GG | GG | GG | GC | 0.611627497 |
| 77 | NO | GG | CC | TT | GG | GG | GG | GC | 0.43629 |
| 78 | NO | GG | CC | CC | GA | GC | GG | GG | 0.258857 |
| 79 | NO | GG | CC | CC | GA | GC | GG | GG | 0.258857 |
| 46 | NO | GG | CT | TT | GA | GG | GG | GC | 0.31 |
| 85 | YES | GG | CC | TT | GG | GG | GG | GG | 0.624 |
| 86 | YES | GG | CC | CC | GG | GG | GG | GG | 0.772176 |
| 87 | YES | GG | CC | CC | GA | GG | GG | GG | 0.527616 |
| 89 | NO | GG | CC | CC | GA | GG | GG | GG | 0.527616 |
| 90 | YES | GG | CC | CC | GG | GG | GG | GG | 0.772175621 |
| 91 | YES | GG | CC | CC | GG | GG | GG | GG | 0.772175621 |
| 94 | YES | GG | CC | CC | GG | GC | GG | CC | 0.403055056 |
| 99 | NO | GG | CC | TT | GG | GG | GG | GG | 0.62401 |
| 93 | NO | GG | CC | CC | GG | GC | GG | GG | 0.59235918 |
| 95 | YES | GG | CC | CC | GA | GG | GG | GG | 0.86178178 |
| 96 | YES | GG | CC | CC | GG | GC | GG | GG | 0.59235918 |
| 97 | NO | GG | CC | CC | GG | GG | GG | GG | 0.772175621 |
| 98 | YES | GG | CC | CC | GA | CC | GG | GC | 0.553983677 |
| 100 | YES | GG | CC | CC | GG | GC | GG | CC | 0.391173048 |
| 101 | YES | GG | CC | CC | GG | GC | GG | CC | 0.391173048 |
| 102 | YES | GG | CC | CC | GA | GC | GG | GG | 0.258857 |
| 103 | YES | GG | CC | TT | GG | GG | GG | GG | 0.624 |
| 104 | YES | GG | CC | TT | GG | GG | GG | GG | 0.624 |
| 105 | NO | GG | CC | CC | GG | GC | GG | CC | 0.391173048 |
| 106 | NO | GG | CC | CC | GG | GG | GG | GC | 0.611627497 |
| 92 | NO | GG | CC | CC | AA | GG | GG | CC | 0.743395056 |
